# Towards objective measurements of habitual dietary intake patterns: comparing NMR metabolomics and food frequency questionnaire data in a population-based cohort

**DOI:** 10.1186/s12937-024-00929-1

**Published:** 2024-03-02

**Authors:** Anna Winkvist, Ingegerd Johansson, Lars Ellegård, Helen M Lindqvist

**Affiliations:** 1 Department of Internal Medicine and Clinical Nutrition, the Sahlgrenska Academy, University of Gothenburg, Box 459, Gothenburg, SE-405 30 Sweden; 2 Department of Public Health and Clinical Medicine, Sustainable Health, Umeå University, Umeå, Sweden; 3 Cariology, Department of Odontology, Umeå University, Umeå, Sweden; 4 Clinical Nutrition Unit, Department of Gastroenterology, Sahlgrenska University Hospital, Gothenburg, Sweden

**Keywords:** Habitual dietary intake, Diet intake patterns, Food frequency questionnaire, NMR metabolomics, Northern Sweden health and disease study

## Abstract

**Background:**

Low-quality, non-diverse diet is a main risk factor for premature death. Accurate measurement of habitual diet is challenging and there is a need for validated objective methods. Blood metabolite patterns reflect direct or enzymatically diet-induced metabolites. Here, we aimed to evaluate associations between blood metabolite patterns and *a priori* and data-driven food intake patterns.

**Methods:**

1, 895 participants in the Northern Sweden Health and Disease Study, a population-based prospective cohort study, were included. Fasting plasma samples were analyzed with ^1^H Nuclear Magnetic Resonance. Food intake data from a 64-item validated food frequency questionnaire were summarized into *a priori* Healthy Diet Score (HDS), relative Mediterranean Diet Score (rMDS) and a set of plant-based diet indices (PDI) as well as data driven clusters from latent class analyses (LCA). Orthogonal projections to latent structures (OPLS) were used to explore clustering patterns of metabolites and their relation to reported dietary intake patterns.

**Results:**

Age, sex, body mass index, education and year of study participation had significant influence on OPLS metabolite models. OPLS models for healthful PDI and LCA-clusters were not significant, whereas for HDS, rMDS, PDI and unhealthful PDI significant models were obtained (CV-ANOVA *p* < 0.001). Still, model statistics were weak and the ability of the models to correctly classify participants into highest and lowest quartiles of rMDS, PDI and unhealthful PDI was poor (50%/78%, 42%/75% and 59%/70%, respectively).

**Conclusion:**

Associations between blood metabolite patterns and *a priori* as well as data-driven food intake patterns were poor. NMR metabolomics may not be sufficiently sensitive to small metabolites that distinguish between complex dietary intake patterns, like lipids.

**Supplementary Information:**

The online version contains supplementary material available at 10.1186/s12937-024-00929-1.

## Introduction

In the latest update of The Global Burden of Disease project from 2020 [1]; low-quality, non-diverse diet was the second (women) or third (men) leading risk factor for premature death. Suboptimal diet is characterized by high intakes of red and processed meat, trans fatty acids and sodium, and low intakes of fruit, vegetables, legumes, whole grains, and nuts and seeds. Unfortunately, the challenge of measuring dietary exposure accurately in free-living individuals remains a limiting step in diet research. Commonly used self-reported methods are all associated with known limitations including misreporting, recall bias and difficulty in assessment of total exposure. Also, variation in individual metabolism due to genetics or the gut microbiome adds complexity to biomarker measurements [2]. Unfortunately, validated objective measurements of overall dietary patterns are few. Consequently, providing accurate and reliable measurements of habitual dietary exposure on large groups of individuals today constitutes one of the most urgent problems in nutrition research [3].

Metabolomics, which is at the downstream end of the post-genomic events, reflects the end products of the genetic, epigenetic and environmental stimuli and their interactions [4]. Thus, the metabolome constitutes a sensitive and precise measure of an organism’s phenotype at a particular time of her/his life. Not surprisingly, the application of metabolomics to nutrition research has expanded rapidly. Still, only a limited number of publications identify metabolite patterns that reflect overall dietary patterns and habitual diets or demonstrate true ability of biomarkers to determine intake and thus to allow classification of people into dietary patterns [5–9]. A persistent challenge is the successful validation and quantification of biomarkers for intakes of proposed dietary patterns [10]. The aim of the current study was to evaluate associations between patterns identified by untargeted metabolomics and by self-reported food intake data from a validated food frequency questionnaire on a sample of participants in a population-based cohort.

## Methods

### Study design and study participants

The Northern Sweden Health and Disease Study, NSHDS, is a biobank with questionnaire data and blood samples from several population-based cohort studies in northern Sweden. The largest cohort is the Västerbotten Intervention Programme, VIP, which started in 1984. The program includes an invitation of all inhabitants in the county of Västerbotten to their regular health care center the year they turn 40, 50 or 60 years of age. For a few years, also 30-years old subjects were invited. Annual participation rate up until today has varied between 50 and 80% of the eligible population. To date, about 60% of the adult population of Västerbotten have participated at least once and an earlier evaluation concluded that there are no indications of systematic bias with respect to socio-demographic characteristics between participants and non-participants [11].

During the health visit, participants complete a questionnaire on lifestyle factors, donate blood samples for research and clinical measurements are collected. Questionnaire data and blood samples are kept by the Unit for Biobank Research, Umeå, Sweden (EBF, https://www.umu.se/en/biobank-research-unit/). VIP is described in detail in Norberg et al. [12].

For the current project, a subsample of 2,000 women and men were selected for detailed evaluation with Nuclear Magnetic Resonance (NMR) untargeted metabolomics. The time window was restricted to the years 2000–2016, because previous research [13] had indicated changes in dietary patterns over time and hence earlier years were excluded. Among visits made by women and men aged > 30 and ≤ 65 years, only those with stored unthawed blood samples and complete questionnaire information on diet, body mass index, smoking and education were considered for sample selection. From this pool, a stratified random sample of 1,000 unique women and 1,000 unique men balanced by 10-year age strata was drawn. Metabolomics analyses were incomplete for five individuals and thus 1,995 individuals were available for further analyses. Outliers with respect to BMI (< 19.0 and > 35.0 kg/m^2^) and fasting plasma glucose levels (> 8.0 mmol/l) exerted strong impact on metabolomics models. Hence, these individuals were removed and the sample size for the final analyses was 1,895 with participation in VIP between the years 2000–2016.

### Metabolomics analyses

Fasting blood samples were stored at -80 °C until analysis, and prepared according to In Vitro Diagnostics Research (IVDr; Bruker BioSpin, Rheinstetten, Germany) standard operating procedures [14]. Daily quality assurance included ensuring that sample temperature (calibration on 99.8% methanol-d_4_), shimming quality and water suppression (2mM sucrose sample in 10% D_2_O) and quantification reference (certified sample containing five metabolites of known concentration) were within specifications. Prior to ^1^H NMR analyses, previously unthawed plasma samples were thawed for 30 min at room temperature and thereafter centrifuged at 3,500x *g* for 1 min at 4 °C. Next, 100 µL plasma was mixed with 100 µL NMR buffer (75 mM Na_2_HPO_4_, 20% *v/v* D_2_O, 0.08% TSP-d_4_, 0.04% NaN_3_, pH 7.4) in a deep well plate (Porvair, cat no 53.219030), with the aid of a SamplePro Tube L liquid handler (Bruker BioSpin). The plate was shaken at 400 r/min at 12 °C for 5 min in a Thermomixer Comfort (Eppendorf). Then, an 180 µL aliquote was transferred to 3 mm SampleJet NMR tubes using the SamplePro L; all sample tubes, the deep well plate and the SampleJet rack were kept at 2 °C until analyses.

All ^1^H NMR spectra were measured on a Bruker 600 MHz Avance III HD spectrometer equipped with a room temperature 5 mm BBI probe and a cooled (6 °C) SampleJet automatic sample changer for sample handling. Here, 1D NOESY (´noesygppr1d´pulse sequence) was used for peak selection and metabolite quantification and 1D CPMG (´cpmgpr1d´) and 2D J-resolved (´jresgpprqf´) spectra, obtained according to the standard IVDr parameter settings at 310 K, were used for manual identification of peaks. TSP-d_4_ was used for referencing.

Sodium phosphate (Na_2_HPO_4_) and sodium azide (NaN_3_) were bought from SigmaAldrich, deuterium oxide (D_2_O) from CortecNet, and 3-(trimethylsilyl) propionic-2,2,3,3-d_4_ acid sodium salt (TSP-d_4_) from MerckMillipore. Data were aligned and peaks were selected by R using ’speaq 2´ [15]. Poor water suppression in several samples influenced the spectra around 4.7 ppm; therefore spectra between 4.2 and 5.2 ppm were not included in the current analyses. However, this did not influence the model quality in any material way. In total 230 peaks between chemical shifts − 0.236–8.096 ppm were included. Annotation of discriminating metabolites selected from the multivariate models was done by Chenomx NMR suite 8.31 (Chenomx Inc.) with the aid of the Human Metabolome Database [16] and an in-house implementation of the STOCSY routine [17].

### Dietary assessment

Participants in VIP filled in a semi-quantitative food frequency questionnaire (FFQ) that consists of 64 questions on common food items and dishes and reflects habitual intake during the last year. Portion sizes were indicated on four pictures with varying portion sizes for meat/fish, staple food and vegetables. Frequency of intake of the food items was indicated on a nine-grade scale from never to ≥ 4 times/day. Frequency of intake was converted to grams per day using the indicated portion sizes as well as natural sizes (e.g., fruit) or either age or gender-specific portion sizes. Daily energy and nutrient intakes were calculated by linking the food intake data to the national food composition database at the Swedish Food Agency (https://soknaringsinnehall.livsmedelsverket.se/). All dietary data in NSHDS are curated as Northern Sweden Diet Database, NSDD.

Originally an 84-item FFQ was designed. This version was validated against ten repeated 24-hour recalls and plasma β-carotene in 246 study participants [18]. Participants also repeated the FFQ twice, one year apart. The results indicated good correlations in energy and nutrient intake between the two occasions and the FFQ was deemed to be of similar quality as that of other prospective cohort studies using FFQ as a method to measure food intake [19]. Further, reported intake of several fatty acids has been validated against 24-hour recalls and fatty acid profile of erythrocyte membranes [20], and reported intake of phytosterols [21]. Later, several similar food groups were collapsed into larger groups, resulting in a 64-item FFQ. This version has been validated against biomarkers for reported intake of B vitamins [22].

For the current analyses, only individuals with reported dietary intake of acceptable quality were included. Inclusions were based on having < 10% missing answers on the FFQ, and food intake level (reported energy intake/calculated basal metabolic rate) within 1–99% of the range for each sex in the entire VIP cohort.

### Construction of a priori and data-driven diet scores and indices

Diet intake patterns have been described for all participants in NSDD previously, using *a priori* scores and indices as well as *a posteriori* data-driven clustering, and these were used in the present analyses. A Healthy Diet Score (HDS) was calculated as previously described [23]. The score is based on intake of eight food and beverage groups. Favorable groups include fish, fruit (except juice), vegetables (except potatoes) and whole grain. Unfavorable groups include red and processed meat, desserts and sweets, sugar-sweetened beverages and fried potatoes. Within each sex, intakes are ranked in ascending quartile ranks for favorable groups and in descending quartile ranks for unfavorable groups. The sum of the quartile ranks yields the score, with a maximum of 24 and higher scores reflecting a healthier diet.

A relative Mediterranean Diet Score (rMDS) was calculated as described by Buckland et al. [24]. The score indicates adherence to a Mediterranean style diet and is based on intake of nine components. Tertiles of intake, expressed as g*1000/kcal*day, were calculated for vegetables excluding potatoes; fruit including nuts and seeds; legumes, fresh and frozen fish excluding fish products and preserved fish, olive oil and cereals. The tertiles were assigned values of 0–2. For total meat and dairy products, similar tertiles were constructed and the scoring was reversed to account for a putative negative effect on health. Alcohol was scored 2 for moderate consumption and 0 for consumption outside of this range. The final score had a maximum of 9, indicating high adherence to a healthy Mediterranean-style diet.

A plant-based diet index, PDI, was developed as described by Satija et al. [25]. Foods were combined into 15 homogeneous groups (healthful plant foods: whole grains, fruits, vegetables, legumes, vegetable oils, coffee/tea; unhealthful plant foods: sweetened beverages, refined grains, potato, sweets/desserts; and animal foods: animal fat, dairy, fish/seafood, poultry/red meat, and miscellaneous animal-based foods). Within each sex, quintiles of frequency of intake/day were constructed. For PDI, participants were assigned 5 points if they were above their fifth quintile of intake of any plant food, 4 points if between the fifth and fourth quintile of intake and so forth down to 1 point if below the first quintile of intake. For animal foods the reverse scoring was used, i.e., participants were assigned 1 point if above their fifth quintile of intake etc. Points for all 15 food groups were summarized to the PDI. Further, a healthful plant diet index was constructed, hPDI. Here, only healthful plant foods were included in the positive ranking (i.e., 5 points if above highest quintile etc.) whereas both unhealthful plant foods and animal foods were included in the reverse ranking (i.e., 1 point if above the highest quintile, etc.). Lastly, an unhealthful plant diet index was constructed, uPDI. Here, unhealthful plant foods were included in the positive ranking whereas healthful plant foods and animal foods were included in the reverse ranking. For all three indices, minimum and maximum values ranged 15 and 75.

Finally, latent class analyses have been applied to NSDD to identify distinct, mutually exclusive latent clusters of habitual diet [13]. Female and male NSDD participants between 2000 and 2007 and 2008–2016 were modelled separately. The reason for the two time periods was indications that dietary intake patterns had changed in Sweden over the years and hence homogeneous patterns over the entire time span were not expected. In the LCA analyses, individuals are predicted to mutually exclusive groups where within-class variance is minimized and between-class variance is maximized. Reported intake per 1,000 kcal of 40 food groups was used as input data. For all four subgroups, four clusters of food consumption were identified as the optimal class solution based on the Bayesian information criteria (BIC), the LL statistics, class size and pattern interpretability. These clusters captured variations in intake of healthy foods such as fruit and vegetables, high-fiber bread and low-fat milk, and less healthy foods such as high-fat dairy, white bread, sugar, jam and cookies. Clusters from Period 1 (years 2000–2007) have been used in the present analyses because too few participants of the current sample were represented in Period 2 for analyses to be meaningful. Broad description of categorizations as well as intake patterns for the indices, scores and clusters are presented in Supplementary Tables S1 and S2.

### Assessment of non-dietary variables

Anthropometric and socio-demographic data were collected at the participants’ nearest health care center [12]. Height in cm and weight in kg were measured in light clothing, without shoes. Body mass index (BMI) was calculated as weight in kg/height in m^2^. Basal metabolic rate was estimated according to the Schofield equation [26]. Physical activity was measured by combining two questions about occupational and leisure time physical activity into the validated Cambridge Index of Physical Activity [27]. Participants were categorized into inactive, moderately inactive, moderately active and active. Information on smoking was categorized into current smoker; former smoker; and never smoker. Educational level was categorized as basic level of 9 years of schooling; high school; and university.

A 5-minute rest preceded the measurements of systolic and diastolic blood pressures. Blood glucose levels were evaluated with the use of a benchtop analyzer after at least 4 h of fasting. Serum cholesterol and triglycerides had been analyzed in a Reflotron benchtop analyzer at the health care centers (in the earlier years) or using an enzymatic routine method at the nearest hospital (from September 1st 2009). Details of the methods are found in Norberg et al. 2010 [12].

### Statistical analyses

Descriptive results for the study sample are presented using mean and standard deviations or medians and quartiles as well as Spearman correlation coefficients. Continuous variables were adjusted for age. These analyses were performed in IBM SPSS Statistics version 28 (IBM Corp.).

All metabolomics multivariate analyses were performed in SIMCA software v.17.0 (Sartorius Stedim Biotech) with data unit variance-scaled and cross validation groups set to 7 (default). Principal component analysis (PCA) was used to explore clustering patterns of observations and outliers. Orthogonal projections to latent structures (OPLS) include not only x-values (metabolite variables i.e. peaks) but also dependent y-values, e.g., additional known factors that may influence models. Included y-values tested in an OPLS-model were participant characteristics such as BMI, age, sex, education, smoking, physical activity, and year of data collection. To select y-values, a cut-off in the cross-validation analysis of variance (CV-ANOVA) of *p* < 0.05 was applied. OPLS models with HDS, rMDS, PDI, hPDI, uPDI and clusters included one at a time as y-value were evaluated to explore clustering patterns of observations for each of these scores/indices/clusters. If significant models were achieved, the models were further explored by including also participant characteristics as y-values. Lastly, OPLS with discriminant analysis (OPLS-DA) was performed for OPLS models that remained significant both with and without the additional y-values included. Here, lowest quartile (Q1) was compared with highest quartile (Q4) of the score/index. The validity of the OPLS-DA model was assessed using permutation tests (*n* = 999). Validated prediction models for performance are presented using the receiver operating characteristic (ROC) curve for OPLS-DA models. Also, to further test model quality, a test set (∼10% of participants) was selected by computerized randomization before any OPLS-DA analysis were performed. OPLS-DA models were run without the test set participants and this was thereafter used to test the models’ ability to predict high or low dietary quality. The cumulative amount of explained variation in the data summarized by the model (R2X[cum] and R2Y[cum]) and the predictive ability of the model (Q2[cum]) are presented. Class discriminating variables (buckets) of interest from OPLS and OPLS-DA models were selected if variables had loading scores − 0.1 ≥ w ≥ 0.1 and if they had among the 30 highest variable influence on projections values to obtain a reasonable number of models, and these were further assessed by univariate analysis. Mann–Whitney U-test was performed to evaluate metabolites driving the separation in OPLS-DA models. To adjust for multiple testing in univariate analysis a False Discorey Rate (FDR) correction was applied; q values < 0.05 were regarded as significant.

## Results

### Characteristics of the participants

Women were evenly spread among the three age categories, whereas there were relatively fewer men in their 40’s (Table 1). Women were predominantly of normal weight and men predominantly overweight. Women exhibited higher levels of physical activity and university degree was more common among women than among men. For both sexes, about half of the participants had never smoked.

**Table 1:** General characteristics of study participants in the Västerbotten Intervention Programme

Variable	Women (*n* = 932)	Men (*n* = 963)	All (*n* = 1,895)
Age (y) %			
*40*	28.1	15.4	21.6
*50*	36.5	42.3	39.4
*60*	35.4	42.4	38.9
BMI (kg/m^2^) %			
*18.5–25.0*	51.0	34.3	42.5
*>25.0–30.0*	37.9	52.0	45.1
*>30.0*	11.2	13.7	12.5
Physical activity^1^%			
*Inactive*	16.0	16.4	16.2
*Moderately inactive*	26.4	31.0	28.8
*Moderately active*	27.4	29.4	28.4
*Active*	29.8	22.8	26.3
*Missing*	0.4	0.4	0.4
Level of education %			
*Basic level, 9 years*	16.4	21.3	18.9
*High school*	49.2	58.5	53.9
*University*	34.4	20.2	27.2
Smoking %			
*Current smoker*	17.2	15.1	16.2
*Former smoker*	33.2	36.2	34.6
*Never smoker*	49.6	48.7	49.2

^1^Physical activity was measured by combining two questions about occupational and leisure time physical activity into the validated Cambridge Index of Physical Activity [27]

BMI, body mass index

Women reported somewhat higher intake of protein and carbohydrates, expressed as percent of total energy intake, than did men (Table 2). Both sexes had similar median HDS and rMDS whereas PDI indices were somewhat higher among men. For both sexes, HDS, rMDS and hPDI showed Spearman correlation coefficients between ρ = 0.453–0.615, indicating good agreement in their expressions of a healthy diet pattern (Supplemental Tables S3-S4). PDI showed correlations between ρ = 0.101- 0.300 with HDS, rMDS and hPDI, whereas uPDI as expected showed negative correlations with the others. Clusters, not being on ordinal scale, were not evaluated for correlation.

**Table 2:** Reported dietary intake of study participants in the Västerbotten Intervention Programme

Variable	Women (*n* = 932)	Men (*n* = 963)	All (*n* = 1,895)
Energy (kcal/d)	1422 ± 422^1,2^	1883 ± 587	1649 ± 559
Protein (g/d)	54.9 ± 18.2	69.0 ± 23.6	61.9 ± 22.2
Protein (E%)	15.5 ± 2.2	14.7 ± 2.3	15.1 ± 2.3
Animal protein (g/d)	39.1 ± 14.9	50.2 ± 19.3	44.5 ± 18.1
Vegetable protein (g/d)	15.8 ± 6.4	18.9 ± 8.0	17.3 ± 7.4
Carbohydrates (g/d)	170.6 ± 59.2	209.3 ± 78.0	189.6 ± 71.7
Carbohydrates (E%)	47.8 ± 7.5	44.2 ± 7.6	46.0 ± 7.8
Fat (g/d)	54.1 ± 19.3	78.8 ± 29.0	66.2 ± 27.5
Fat (E%)	34.3 ± 6.9	37.7 ± 7.2	36.0 ± 7.3
Fibre (g/d)	17.3 ± 6.9	18.3 ± 8.1	17.8 ± 7.6
PUFA (g/d)	9.0 ± 4.1	12.8 ± 6.4	10.9 ± 5.7
HDS	12.0 (9.0, 15.0)	12.0 (9.0, 14.0)	12.0 (9.0, 15.0)
rMDS PDI hPDI uPDI	9.0 (7.0, 11.0) 47.0 (43.0, 51.0) 45.0 (41.0, 49.0) 44.0 (39.0, 48.0)	9.0 (7.0, 11.0) 48.0 (45.0, 52.0) 45.0 (41.0, 50.0) 45.0 (40.0, 49.0)	9.0 (7.0, 11.0) 48.0 (44.0, 51.0) 45.0 (41.0, 49.0) 44.0 (39.0, 49.0)

^1^Mean and standard deviation, or median and interquartile ranges

^2^Continuous variables adjusted for age

PUFA, poly-unsaturated fatty acids; HDS, Healthy Diet Score; rMDS, relative Mediterranean Diet Score; PDI, plant based diet index; hPDI, healthful plant based diet index; uPDI, unhealthful plant based diet index

### Associations between metabolomics data and background variables

**Fig. 1:**
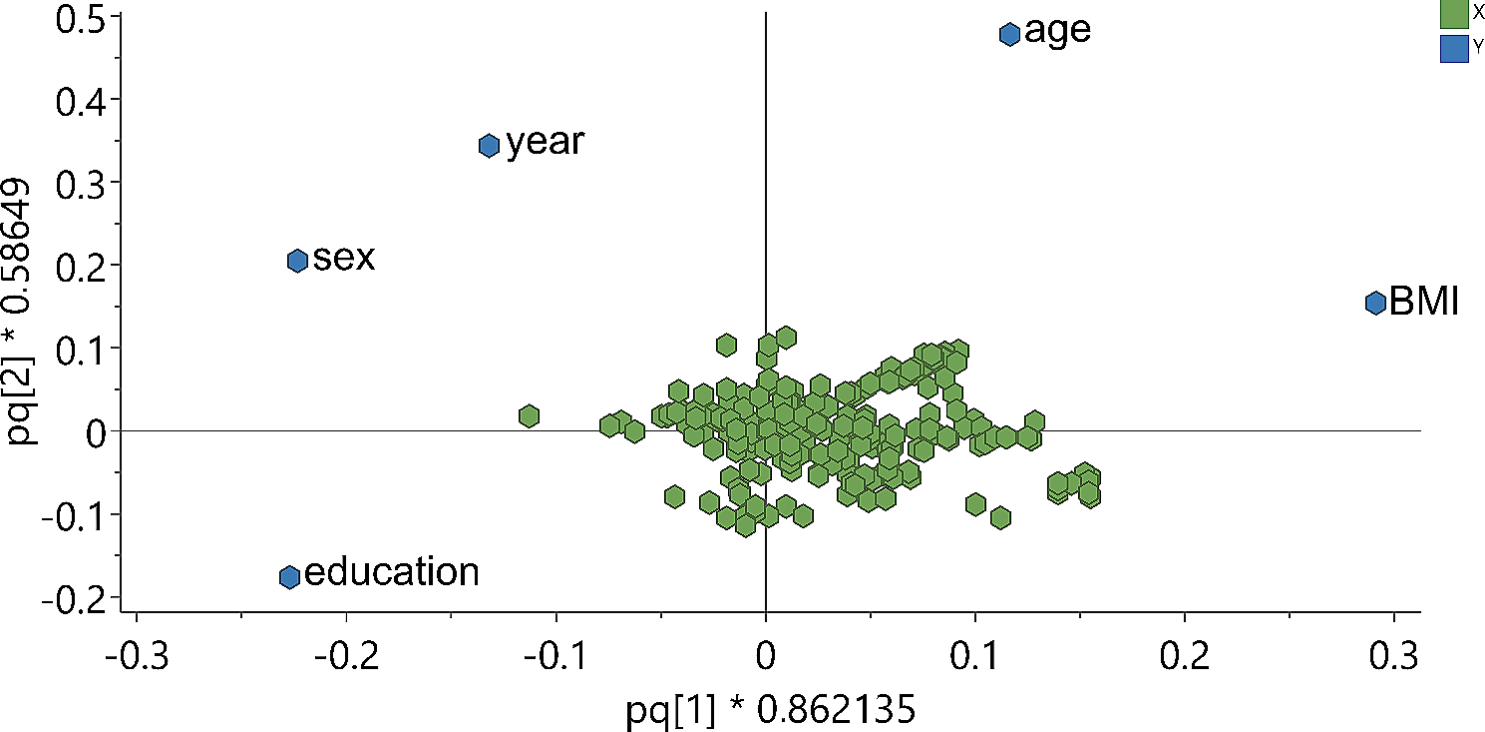
Scores plot for OPLS model of metabolites and background variables. To the left higher education and female sex and to the right higher body mass index (BMI). X-axes, first predictive component; Y-axes, second predictive component. Each circle represents one participant

PCA did not yield any clear clustering patterns among the metabolites (Table 3). Next, an OPLS model was fitted to explore associations between metabolomics data and participant characteristics to evaluate impact of these characteristics on the models. Among the background variables, age, sex, BMI, education and screening year of participation in the study were significantly influential in the OPLS model (*p* < 0.00001 for CV-ANOVA), whereas physical activity and smoking were not (Table 3). The first predictive component of the OPLS model was influenced by high BMI in one direction, and by high education and female gender in the other direction (Fig. 1). The second predictive component of the OPLS model was influenced by high BMI, female gender, high age and more recent year of study participation in one direction, and of high education in the other direction.

**Table 3:** OPLS model statistics from analyses of data from 1,895 participants in the Västerbotten Intervention Programme

Model	Nr of Lv^1^	n	R2X[cum]^2^	R2Y[cum]^3^	Q2[cum]^4^	CV-ANOVA^5^ (p-value)	ROC AUC^6^	Permutation test (Q2)^7^	Classification (Q1/Q4)
PCA-X	7	1,895	0.408		0.318				
OPLS Back-ground data^8^	4 + 1 + 0	1,895	0.279	0.204	0.163	< 0.00001			
OPLS rMDS^9^	1 + 1 + 0	1,895	0.160	0.0794	-0.0372	< 0.0000001			
OPLS-DA rMDS Q1 vs. Q4	1 + 1 + 0	1002	0.184	0.122	0.0472	< 0.0000001	0.71/0.71	0.0663^14^	50%/78%
OPLS HDS^10^	1 + 0 + 0	1,895	0.148	0.0194	0.00924	0.00015			
OPLS PDI^11^	1 + 0 + 0	1,895	0.184	0.0608	0.021	< 0.0000001			
OPLS-DA PDI Q1 vs. Q4	1 + 0 + 0	913	0.0450	0.0664	0.00473	1	0.65/0.65	-0.0340^14^	42%/75%
OPLS hPDI^12^	1 + 0 + 0	1,895	0.100	0.0206	-0.000529	0.116			
OPLS uPDI^13^	1 + 1 + 0	1,895	0.199	0.0776	0.0388	< 0.0000001			
OPLS-DA uPDI Q1 vs. Q4	1 + 1 + 0	993	0.186	0.144	0.0589	< 0.0000001	0.72/0.72	-0.0742^14^	59%/70%
OPLS men time period 1 clusters	1 + 0 + 0	799	0.120	0.0381	-0.000576	1			
OPLS women time period 1 clusters	0 + 0 + 0	668							

1 Latent Variables; 2 Cumulative fraction of the sum of squares of X explained by the selected latent variables; 3 Cumulative fraction of the sum of squares of Y explained by the selected latent variables; 4 Cumulative fraction of the sum of squares predicted by the selected latent variables, estimated by cross-validation; 5 Analysis Of Variance testing of Cross-Validated predictive results; 6 Receiver Operating Area under the curve; 7 the intercept between real and random models, degree of overfit. 8 y-values included BMI, age, time, sex, education, 9 y-value included MDS, 10 y-value included HDS, 11 y-value included PDI, 12 y-value included hPDI, 13 y-value included uPDI, 14 High quality permutation test

*Abbreviations*: PCA, principal component analysis; OPLS, orthogonal projections to latent structures; OPLS-DA, orthogonal projections to latent structures discriminant analyses; BMI, body mass index; DII, Diet Inflammatory Index; rMDS, relative Mediterranean Diet Score; HDS, Healthy Diet Score; Q, first quartile; Q4, fourth quartile; PDI, plant based diet index; hPDI, healthful plant based diet index; uPDI, unhealthful plant based diet index

### Associations between metabolomics patterns and *a priori* diet scores and indices

OPLS model with the *a priori* HDS exhibited poor fit, as indicated by model statistics and by no longer significant p-value for CV-ANOVA when all significant y-values (age, sex, BMI, education, and screening year of participation) were included in the OPLS model; this model was not further elaborated. OPLS model with *a priori* rMDS exhibited a slightly better fit, as indicated by significant CV-ANOVA, R2 × 0.160, R2Y 0.079, and Q2 0.037. An OPLS-DA model was fitted for the two first predictive components with the lowest quartile vs. the highest quartile of rMDS (Table 3). The ROC areas under the curve were 71% and the ability of the model to correctly predict Q1 and Q4 for rMDS was 50%/78%.

**Fig. 2a:**
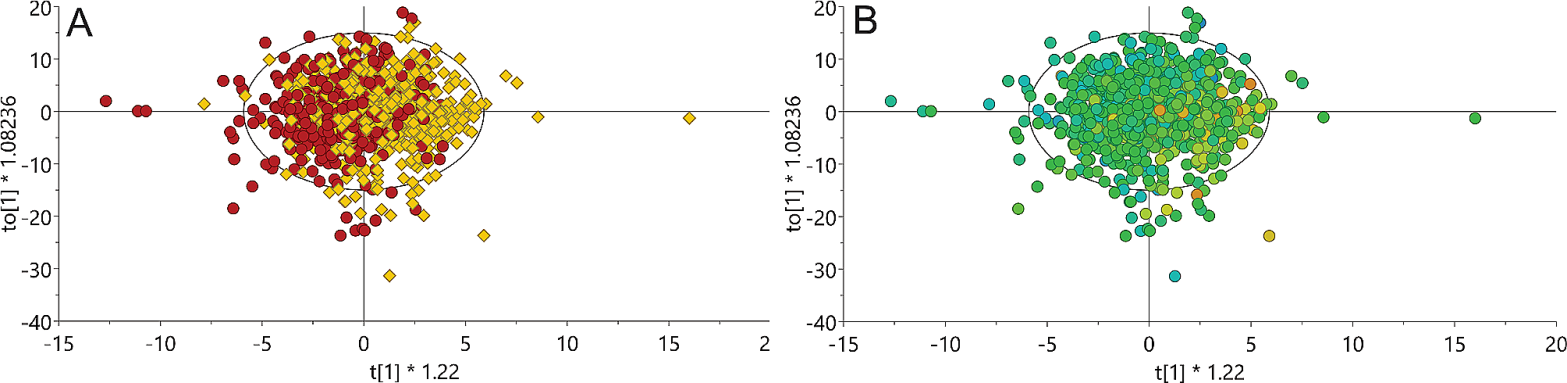
Results from OPLS-DA model of uPDI. Red circles represent quartile 4 and yellow triangles represent quartile 1. **2b** OPLS-DA model of uPDI colored by HDL-concentrations (low HDL, blue and high HDL, orange). X-axes, first predictive component explaining the variation between groups; Y-axes, second predictive component explaining the variation within groups

OPLS models with PDI and uPDI also exhibited a decent fit, as indicated by significant CV-ANOVA and R2 × 0.184, R2Y 0.061 and Q2 0.021 for PDI, and R2 × 0.199, R2Y 0.078 and Q2 0.000529 for uPDI (Table 3). Surprisingly, OPLS model for hPDI exhibited poor fit (CV-ANOVA non-significant). OPLS-DA models were therefore fitted for PDI and uPDI, for the two first predictive components with the lowest quartile vs. the highest quartile of the indices (Table 3; Fig. 2 for uPDI).

The ROC areas under the curve were 65% or more for both indices (Table 3). The ability of the models to correctly predict Q1 and Q4 for PDI was 42%/75% and for uPDI 59%/70%.

### Associations between metabolomics data and data-driven diet intake clusters

OPLS models were run with the inductively generated diet intake clusters included. These clusters had previously been generated among women and men separately, and therefore the OPLS models were run for each sex separately. No obvious patterns with respect to clusters were identified and no further modelling was performed (Table 3).

### Metabolites that discriminated between different intake patterns

Metabolites that discriminated between different intake patterns in the models shown in Table 3 were further inspected. Those that had a loading score w > ± 0.1 and that were among the top 30 influential variables based on projections scores were tested with univariate analyses (Mann-Whitney U-test). All selected metabolites were significantly different between Q1 and Q4 of the respective intake patterns in univariate tests also after FDR (Table 4). For PDI, 1,5-anhydrosorbitol + serine and glutamine were higher in Q4 than in Q1, and 2-hydroxyvalerate and 3-hydroxybutyrate were lower. For uPDI, participants in Q4 had higher 1,5-anhydrosorbitol, lactate and several of the lipid compartments and a lower acetate concentration compared to those in Q1. Also for rMDS, 1,5-anhydrosorbitol and lactate were found to discriminate, but in the opposite direction of the findings for uPDI, indicating that these two scores capture similar dietary pattern.

**Table 4:** Univariate statistics for metabolites that discriminated significantly between quartiles 1 (Q1) and 4 (Q4) in OPLS-DA models for relative Mediterranean Diet Score, Plant based Diet Index and Unhealthy Plant based Diet Index

Metabolite^1^	^1^H chemical shift region	rMediterranean Diet Score			Plant based Diet Index			Unhealthful Plant based Diet Index				
		Q1	Q4	q^2^	Q1	Q4	q^2^	Q1	Q4	q^2^		
1,5-anhydrosorbitol	3.893	↑	↓	1.46E-3				↓	↑	2.55E-11		
1,5-anhydrosorbitol + serine	3.996				↓	↑	0.0300					
1,5-anhydrosorbitol + serine	4.005							↓	↑	6.69E-5		
1,5-anhydrosorbitol + unidentified	3.367	↑	↓	2.20E-9				↓	↑	6.51E-9		
2-hydroxyvalerate	0.931				↑	↓	3.02E-3					
3-hydroxybutyrate	2.363				↑	↓	0.0120					
acetate	1.935							↑	↓	1.35E-14		
glutamine	2.454				↓	↑	2.17E-3					
glutamine + unidentified	2.119				↓	↑	3.81E-3					
lactate	1.34	↑	↓	2.34E-4				↓	↑	5.21E-3		
lactate + proline	4.147	↑	↓	2.32E-4				↓	↑	3.05E-4		
lipid	1.29							↓	↑	8.87E-3		
NAAs/fatty acyl allylic protons from lipoprotein lipids	2.018							↓	↑	9.78E-3		
phosphocholines, phosphoethanolamine + unidentified	3.239							↑	↓	2.10E-7		

1 Mean chemical shift (ppm) for the bucket used for Mann-Whitney U-test. Selected if variables had loading scores − 0.1 ≥ w ≥ 0.1 and if they had among the 30 highest variable influence on projections values. 2 Mann-Whitney U-test. Significant Mann-Whitney U-test after FDR correction for 19 univariate tests (q < 0.05). ↑ = high, ↓ = low

*Abbreviations*: Q1, first quartile; Q4, last quartile

## Discussion

Associations between inductively created clusters of metabolites identified with NMR metabolomics and *a priori* diet pattern scores and indices and inductively created dietary intake clusters were evaluated. Participants represented a large population-based cohort, and the dietary information was collected with a validated FFQ reflecting habitual intake. NMR metabolomics models were not able to predict dietary intake clusters and they showed poor association with HDS and hPDI. Somewhat better model fits were obtained for rMDS, PDI and uPDI although model qualities were not impressive.

Accurate measurement of habitual diet is challenging and there is a need for validated objective methods. Blood metabolite patterns reflect direct or enzymatically diet-induced metabolites and thus may capture immediate responses to exposures, in contrast to the field of genomics. Hence, there is today great interest in evaluating agreement between blood metabolite patterns and habitual food intake patterns. Still, for metabolomics to be useful in large epidemiological studies, putative biomarkers have to reliably reflect habitual intake also when only one biological sample per individual is available. Previous research has shown this to be the case: Floegel and colleagues [28] used repeated fasting serum samples collected 4 months apart and demonstrated that reliability for most of the 163 metabolites evaluated was good. The authors concluded that for most metabolites a single measurement is sufficient to assess long-term exposure in large epidemiological studies. Finally, urine samples have higher concentrations and wider range of food-derived compounds, except most lipid-soluble compounds, than has blood which is under homeostatic control. Hence, urine may be preferred for identifying biomarkers of food intake. Even so, in many large epidemiological studies blood samples and not urine samples are available.

A recent review summarized biomarkers of diet patterns evaluated in smaller controlled intervention studies [6]. Most of the identified 30 studies used MS techniques but a handful used NMR technique like in our study. Many studies applied targeted metabolomics in search for known biomarkers, e.g., n-3 index, 24-h urinary electrolytes and carotenoids. Some studies were exploratory and the most commonly discovered biomarkers were those associated with intake of fish, protein and lipids, but also meat, vegetables, fruit, dairy, chocolate, vitamins, whole grains and legumes. The review concluded that most biomarkers were associated with specific foods or nutritional aspects of the diet but, because these foods appear in many diet patterns, the biomarkers lacked specificity for a particular dietary pattern. The review also pointed out the challenge to compare results across studies that use different analytical platforms; when metabolites were investigated within the same study with both MS and NMR techniques, only one overlapping metabolite was identified. Hence, comparisons of our results with those from studies using other metabolomics platforms, and urine instead of serum as biofluid, should be made with caution [6]. Another recent review of metabolomic biomarkers of healthy dietary patterns reported that metabolites associated with vegetarian diets were amino acids (emphasized in NMR metabolomics), whereas metabolites associated with the Mediterranean diet were lipids (emphasized in MS metabolomics) [9]. The authors likewise caution about comparing studies using different metabolomics platforms.

Only a few studies have evaluated habitual dietary patterns in larger cohorts, like ours. O’Sullivan and colleagues used NMR metabolomics but applied to urine samples [29]. Metabolites responsible for separation of clusters included TMAO, glycine, *O*-acetylcarnitine and phenylacetylglutamine, thus mainly reflecting red meat and vegetable intake. A study using NMR metabolomics on a smaller sample compared intake data from repeated 24 h recalls with metabolites in urine samples [30]. Here, metabolomics models were able to predict adherence to healthy diets as captured by Nutrient Rich Food index, DASH diet and OMNIHEART. Perhaps associations between metabolomics patterns and dietary intake patterns are stronger when metabolites are compared with indices based on nutrient content rather than on food content, because of the heterogeneous content of macro-and micronutrients in foods. Several researchers have used serum samples like our study but applied MS metabolomics [10, 31]. Here, associations have been found between dietary indices and scores and metabolites such as fatty acids profiles and amino acids. With respect to food intake patterns, most metabolites have reflected intake of fish, fruits, vegetables, alcohol and whole grains; i.e., as for the evaluations of smaller controlled intervention studies, metabolite patterns were specific for certain foods but not for dietary patterns per se.

We have previously shown that NMR metabolomics has the ability to distinguish between habitual meat and nonmeat consumers (97.5% correctly classified using serum samples and 91% correctly classified using urine samples), but lower ability to distinguish between habitual vegans and nonvegans (92.5% correctly classified using serum samples and 75% correctly classified using urine samples) [32, 33]. Here, most of the discriminating metabolites were related to amino acids. This likely explains the poorer ability of NMR metabolomics than of MS metabolomics to separate dietary intake patterns beyond meat vs. no meat, at least for dietary intake patterns based on food content rather than on nutrient content.

Plant-based dietary patterns have been associated with lower risk of cardiovascular diseases [34] and it is therefore important to identify these dietary patterns in research on diet and health. Recent comparisons of the PDI indices and metabolites in plasma using MS among Danish [35] and American cohorts [36] found a minor set of metabolites that were specific for each index. In our analyses using NMR metabolomics, glutamine was one of the discriminating metabolites that in the PDI-model was higher in Q4 than in Q1. Glutamine has been found to be higher in individuals with diets that exclude meat and other animal-based foods [32], and thus have a higher intake of plant food. 2-hydroxyvalerate, a metabolite found in meat and produced endogenously, was lower in Q4 than in Q1 for PDI, and this can possibly indicate a lower intake of meat. 3-hydroxybutyrate, a keton body and metabolite from branched chain amino acids, also was lower in Q4 than in Q1 for PDI. The strongest OPLS-DA models were obtained when comparing Q1 and Q4 for rMDS and uPDI.

For rMDS, Q4 was associated with a lower concentration of 1,5-anhydrosorbitol than Q1. This metabolite is a validated marker of short-term glycemic control. In addition, the lactate concentration was low in Q4, and high concentrations have previously been reported in metabolically impaired subjects [37]. The opposite was seen for uPDI, which is an index constructed so that a higher score results from consumption of unhealthful plant-based foods such as fruit juice, refined grains, sugar-sweetened soda, potatoes, desserts, and sweets. At last, in uPDI acetate, a short-chain fatty acid, was lower in Q4 than in Q1. Acetate can be produced by gut bacteria but evidence whether serum acetate increases after increased dietary fiber intake are inconclusive [38]. Studies have reported that acetate is higher in type 2 diabetes patients than in healthy subjects [33]. Reduction in weight also has been associated with increased serum acetate [39]. In sum, the metabolites discriminating between uPDI Q1 and Q4 do not seem to be markers of certain foods, but rather markers of consequences of unhealthy eating.

Compared with the more sensitive mass spectrometer (MS)-based metabolomics, NMR is not able to detect low-concentration metabolites and thus has poorer ability to capture compounds such as lipids, fibers and vitamins. This may explain some of the poor associations between our metabolomics patterns and healthy vs. unhealthy dietary intake patterns. However, reasons for using NMR metabolomics in dietary studies are minimal sample preparation, rapid analysis of high reproducibility, reliable metabolite identification, ability to quantify metabolites and low cost [8]. It is therefore important to evaluate the ability of NMR metabolomics to serve as a biomarker of habitual diet for use in large epidemiological studies. Further, for personalized nutrition strategies, NMR has been pointed out as the optimal technical platform because of its technical reliability and affordability [40]. A healthy diet usually refers to low intakes of red and processed meat, trans fatty acids and sodium, and high intakes of fruit, vegetables, legumes, whole grains, and nuts and seeds [1]. How intakes of dairy, potatoe, plant oils like palm oil and alcohol should be classified is debated and varies between different definitions and indicators, as illustrated by the indexes used in this project. This may further explain different results in different studies.

The scores and indices evaluated in this study capture healthy diets in slightly different ways. In the rMDS, higher scores are assigned to high intakes of vegetables (excluding potatoes), fruit, legumes, fish, olive oil, cereals and moderate alcohol intake. Lower scores are assigned to high intakes of total meat and dairy products. Intakes of all components are energy adjusted before individuals are ranked into tertiles. HDS does not include potatoes, juices, legumes or alcohol among beneficial foods, and it does not include dairy or poultry among unfavorable foods. Also, there is no energy adjustment before ranking individuals on intake. Lastly, the PDI simply divides food intake into those of vegetable origin and those of animal origin, regardless of associations with health outcomes. Hence, refined grains, sodas and sweets and desserts receive positive scores and only foods of animal origin receive reverse scores. hPDI distinguishes between health aspects and only assigns positive scores to healthful foods of vegetable origin and reverse scores for unhealthful foods of vegetable origin as well as for animal foods. Finally, uPDI is an anomaly that assigns positive scores to the unhealthful foods of vegetable origin and reverse scores to all other foods. No adjustment for energy intake is made when creating the PDI indices. Negative correlations between uPDI and the other scores and indices, and the positive correlations among rMDS, HDS and hPDI, are therefore expected. Further, each score and index represent different combinations of amino acids, lipids and carbohydrates and these are detected by NMR technique to different extents. Hence, it is no surprise that a comparison between each score or index and detected metabolites yields somewhat different results.

Postmenopausal status of the women was not measured and may have affected metabolite patterns among women in the older age group that we were unable to explain. We used fasting blood samples and not postprandial blood samples; the former are more influenced by background characteristics as serum concentrations are controlled by homeostasis and reflect exogenous as well as endogenous processes, whereas the latter show stronger traces of food metabolites. Hence, weaker associations between diet intake data and circulating metabolites are expected from epidemiological studies than from intervention studies [7]. Still, biomarkers that have been identified in cohort and case-referent studies have proven to be more sensitive and robust, perhaps because they are detectable in spite of metabolite degradation during storage [7]. Regardless of which study design is optimal for comparison with metabolomics data, the aim of the current project was to identify objective biomarkers of habitual dietary intake.

The FFQ consisted of 64 food items, decided upon in 1985 and unchanged since to maintain continuity in data collection. Hence, it is a somewhat crude estimate of the diet diversity of inhabitants in Västerbotten due to the limited number of food items included and because it lacks modern products such as vegan alternatives. Nuts and seeds are not captured, and fruits and vegetables are only captured by a few questions. During 2020–2022 a new updated and extended digital version has been developed that addresses these issues, but the dietary data used for the current analyses suffer from the limitations of the older version. Also, the original version contained 84 food items that were later reduced to 64 items mostly by combining similar food stuffs. Most validations were carried out on the 84-item version but there is little reason to believe that these results do not also apply to the 64 version.

Strengths of the presented analyses include the large sample size for a metabolomics project and that subjects originated from a large, population-based cohort that has been well characterized over time. We hence believe that the results can be generalized to populations with similar Western diets. Diet scores, indices and clusters were created within the entire NSHDS database with over 120 000 participants, yielding robust estimates of these variables as they reflect relative positions within the sample in which they were created. The FFQ has been validated, blood was donated concurrently with questionnaire data and blood samples have been stored at -80 degrees. Limitations include that the FFQ only included 64 food items, the inherent measurement bias with all subjective dietary intake tools, and that NMR metabolomics only detects somewhat larger metabolites and thus has poorer ability to capture lipids, fibers and vitamins in the diet.

## Conclusions

Associations between blood metabolite patterns and a priori as well as data-driven food intake patterns were poor. NMR metabolomics may not be sufficiently sensitive to metabolites that distinguish between complex dietary intake patterns, for example lipids. There is a need for intervention studies over longer periods of time where several levels of intake aligned with different dietary intake patterns are experimented with, and different metabolomics techniques evaluated.

### Electronic supplementary material

Below is the link to the electronic supplementary material.

Supplementary Material 1

## Data Availability

Data on dietary intake and background variables described in the manuscript, codebook, and analytic code will not be made available due to legal limitations. Metabolomics data may be obtained from the authors.

## Abbreviations

ANOVA: Analyses of Variance
BIC: Bayesian information criteria
BMI: Body Mass Index
BMR: Basal Metabolic Rate
HDS: Healthy Diet Score
FFQ: Food Frequency Questionnaire
hPDI: Healthful Plant Based Diet Index
NMR: Nuclear Magnetic Resonance
NSHDS: Northern Sweden Health and Disease Study
NSDD: Northern Sweden Dietary Data
PCA: Principal Component Analysis
OPLS: Orthogonal Projections to Latent Structures
OPLS: DA–Orthogonal Projections to Latent Structures Discriminant Analyses
PDI: Plant Based Diet Index
PUFA: Poly–Unsaturated Fatty Acids
rMDS: Relative Mediterranean Diet Score
ROC: Receiver Operating Characteristic
uPDI: Unhealthful Plant Based Diet Index
VIP: Västerbotten Intervention Programme

## References

[1] Roth GA, Mensah GA, Johnson CO, Addolorato G, Ammirati E, Baddour LM, Barengo NC, Beaton AZ, Benjamin EJ, Benziger CP (2020). Global Burden of Cardiovascular diseases and Risk factors, 1990–2019: Update from the GBD 2019 study. J Am Coll Cardiol.

[2] Maruvada P, Lampe JW, Wishart DS, Barupal D, Chester DN, Dodd D, Djoumbou-Feunang Y, Dorrestein PC, Dragsted LO, Draper J (2020). Perspective: dietary biomarkers of intake and exposure-exploration with Omics approaches. Adv Nutr.

[3] Gao Q, Praticò G, Scalbert A, Vergères G, Kolehmainen M, Manach C, Brennan L, Afman LA, Wishart DS, Andres-Lacueva C (2017). A scheme for a flexible classification of dietary and health biomarkers. Genes Nutr.

[4] Bertram HC, Jakobsen LMA (2018). Nutrimetabolomics: integrating metabolomics in nutrition to disentangle intake of animal-based foods. Metabolomics.

[5] Dragsted LO, Gao Q, Scalbert A, Vergères G, Kolehmainen M, Manach C, Brennan L, Afman LA, Wishart DS, Andres Lacueva C (2018). Validation of biomarkers of food intake-critical assessment of candidate biomarkers. Genes Nutr.

[6] Liang S, Nasir RF, Bell-Anderson KS, Toniutti CA, O’Leary FM, Skilton MR (2022). Biomarkers of dietary patterns: a systematic review of randomized controlled trials. Nutr Rev.

[7] Playdon MC, Moore SC, Derkach A, Reedy J, Subar AF, Sampson JN, Albanes D, Gu F, Kontto J, Lassale C (2017). Identifying biomarkers of dietary patterns by using metabolomics. Am J Clin Nutr.

[8] Naureen Z, Cristoni S, Donato K, Medori MC, Samaja M, Herbst KL, Aquilanti B, Velluti V, Matera G, Fioretti F (2022). Metabolomics application for the design of an optimal diet. J Prev Med Hyg.

[9] Kim H, Rebholz CM (2021). Metabolomic biomarkers of healthy dietary patterns and Cardiovascular outcomes. Curr Atheroscler Rep.

[10] Noerman S, Landberg R (2023). Blood metabolite profiles linking dietary patterns with health-toward precision nutrition. J Intern Med.

[11] Weinehall L, Hallgren CG, Westman G, Janlert U, Wall S (1998). Reduction of selection bias in primary prevention of cardiovascular disease through involvement of primary health care. Scand J Prim Health Care.

[12] Norberg M, Wall S, Boman K, Weinehall L. The Västerbotten intervention Programme: background, design and implications. Glob Health Action 2010, 3. 10.3402/gha.v3i0.4643 PMC2844807 20339479

[13] Huseinovic E, Hörnell A, Johansson I, Esberg A, Lindahl B, Winkvist A (2019). Changes in food intake patterns during 2000–2007 and 2008–2016 in the population-based Northern Sweden Diet Database. Nutr J.

[14] Dona AC, Jiménez B, Schäfer H, Humpfer E, Spraul M, Lewis MR, Pearce JT, Holmes E, Lindon JC, Nicholson JK (2014). Precision high-throughput proton NMR spectroscopy of human urine, serum, and plasma for large-scale metabolic phenotyping. Anal Chem.

[15] Beirnaert C, Meysman P, Vu TN, Hermans N, Apers S, Pieters L, Covaci A, Laukens K (2018). Speaq 2.0: a complete workflow for high-throughput 1D NMR spectra processing and quantification. PLoS Comput Biol.

[16] Wishart DS, Jewison T, Guo AC, Wilson M, Knox C, Liu Y, Djoumbou Y, Mandal R, Aziat F, Dong E (2013). HMDB 3.0–The human metabolome database in 2013. Nucleic Acids Res.

[17] Cloarec O, Dumas ME, Craig A, Barton RH, Trygg J, Hudson J, Blancher C, Gauguier D, Lindon JC, Holmes E, Nicholson J (2005). Statistical total correlation spectroscopy: an exploratory approach for latent biomarker identification from metabolic 1H NMR data sets. Anal Chem.

[18] Johansson I, Hallmans G, Wikman A, Biessy C, Riboli E, Kaaks R (2002). Validation and calibration of food-frequency questionnaire measurements in the Northern Sweden Health and Disease cohort. Public Health Nutr.

[19] Johansson G, Wikman A, Ahrén AM, Hallmans G, Johansson I (2001). Underreporting of energy intake in repeated 24-hour recalls related to gender, age, weight status, day of interview, educational level, reported food intake, smoking habits and area of living. Public Health Nutr.

[20] Wennberg M, Vessby B, Johansson I (2009). Evaluation of relative intake of fatty acids according to the Northern Sweden FFQ with fatty acid levels in erythrocyte membranes as biomarkers. Public Health Nutr.

[21] Klingberg S, Winkvist A, Hallmans G, Johansson I (2013). Evaluation of plant sterol intake estimated with the Northern Sweden FFQ. Public Health Nutr.

[22] Johansson I, Van Guelpen B, Hultdin J, Johansson M, Hallmans G, Stattin P (2010). Validity of food frequency questionnaire estimated intakes of folate and other B vitamins in a region without folic acid fortification. Eur J Clin Nutr.

[23] Nettleton JA, Hivert MF, Lemaitre RN, McKeown NM, Mozaffarian D, Tanaka T, Wojczynski MK, Hruby A, Djoussé L, Ngwa JS (2013). Meta-analysis investigating associations between healthy diet and fasting glucose and insulin levels and modification by loci associated with glucose homeostasis in data from 15 cohorts. Am J Epidemiol.

[24] Buckland G, Agudo A, Luján L, Jakszyn P, Bueno-de-Mesquita HB, Palli D, Boeing H, Carneiro F, Krogh V, Sacerdote C (2010). Adherence to a Mediterranean diet and risk of gastric adenocarcinoma within the European prospective investigation into Cancer and Nutrition (EPIC) cohort study. Am J Clin Nutr.

[25] Satija A, Bhupathiraju SN, Rimm EB, Spiegelman D, Chiuve SE, Borgi L, Willett WC, Manson JE, Sun Q, Hu FB (2016). Plant-based dietary patterns and incidence of type 2 diabetes in US men and women: results from three prospective cohort studies. PLoS Med.

[26] Schofield WN, Schofield C, James WPT. Basal metabolic rate. Human Nutrition Clinical Nutrition 1985, 39 Supplement 1:1–96.

[27] Peters T, Brage S, Westgate K, Franks PW, Gradmark A, Tormo Diaz MJ, Huerta JM, Bendinelli B, Vigl M, Boeing H (2012). Validity of a short questionnaire to assess physical activity in 10 European countries. Eur J Epidemiol.

[28] Floegel A, Drogan D, Wang-Sattler R, Prehn C, Illig T, Adamski J, Joost HG, Boeing H, Pischon T (2011). Reliability of serum metabolite concentrations over a 4-month period using a targeted metabolomic approach. PLoS ONE.

[29] O’Sullivan A, Gibney MJ, Brennan L (2011). Dietary intake patterns are reflected in metabolomic profiles: potential role in dietary assessment studies. Am J Clin Nutr.

[30] Posma JM, Garcia-Perez I, Frost G, Aljuraiban GS, Chan Q, Van Horn L, Daviglus M, Stamler J, Holmes E, Elliott P, Nicholson JK (2020). Nutriome-metabolome relationships provide insights into dietary intake and metabolism. Nat Food.

[31] Altmaier E, Kastenmüller G, Römisch-Margl W, Thorand B, Weinberger KM, Illig T, Adamski J, Döring A, Suhre K (2011). Questionnaire-based self-reported nutrition habits associate with serum metabolism as revealed by quantitative targeted metabolomics. Eur J Epidemiol.

[32] Lindqvist HM, Rådjursöga M, Malmodin D, Winkvist A, Ellegård L (2019). Serum metabolite profiles of habitual diet: evaluation by 1H-nuclear magnetic resonance analysis. Am J Clin Nutr.

[33] Lindqvist HM, Rådjursöga M, Torstensson T, Jansson L, Ellegård L, Winkvist A (2021). Urine metabolite profiles and nutrient intake based on 4-Day weighed Food Diary in Habitual vegans, vegetarians, and omnivores. J Nutr.

[34] Gan ZH, Cheong HC, Tu YK, Kuo PH. Association between Plant-Based Dietary Patterns and Risk of Cardiovascular Disease: A Systematic Review and Meta-Analysis of Prospective Cohort Studies. Nutrients 2021, 13. 10.3390/nu13113952 PMC8624676 34836208

[35] Lanuza F, Meroño T, Zamora-Ros R, Bondonno NP, Rostgaard-Hansen AL, Sánchez-Pla A, Miro B, Carmona-Pontaque F, Riccardi G, Tjønneland A (2023). Plasma metabolomic profiles of plant-based dietary indices reveal potential pathways for metabolic syndrome associations. Atherosclerosis.

[36] Wang F, Baden MY, Guasch-Ferré M, Wittenbecher C, Li J, Li Y, Wan Y, Bhupathiraju SN, Tobias DK, Clish CB (2022). Plasma metabolite profiles related to plant-based diets and the risk of type 2 diabetes. Diabetologia.

[37] Jones TE, Pories WJ, Houmard JA, Tanner CJ, Zheng D, Zou K, Coen PM, Goodpaster BH, Kraus WE, Dohm GL (2019). Plasma lactate as a marker of metabolic health: implications of elevated lactate for impairment of aerobic metabolism in the metabolic syndrome. Surgery.

[38] Vinelli V, Biscotti P, Martini D, Del Bo C, Marino M, Meroño T, Nikoloudaki O, Calabrese FM, Turroni S, Taverniti V et al. Effects of Dietary Fibers on Short-Chain Fatty Acids and Gut Microbiota Composition in Healthy Adults: A Systematic Review. Nutrients: 2022, 14. 10.3390/nu14132559 PMC9268559 35807739

[39] González Hernández MA, Canfora EE, Pasmans K, Astrup A, Saris WHM, Blaak EE. The Relationship between Circulating Acetate and Human Insulin Resistance before and after Weight Loss in the DiOGenes Study. Nutrients 2020, 12. 10.3390/nu12020339 PMC7071284 32012996

[40] Keijer J, Escoté X, Galmés S, Palou-March A, Serra F, Aldubayan MA, Pigsborg K, Magkos F, Baker EJ, Calder PC et al. Omics biomarkers and an approach for their practical implementation to delineate health status for personalized nutrition strategies. Crit Rev Food Sci Nutr 2023:1–29. 10.1080/10408398.2023.2198605 37077157

