# Supplementary material for: Towards objective measurements of habitual dietary intake patterns: comparing NMR metabolomics and food frequency questionnaire data in a population-based cohort
Source: Nutr J. 2024 Mar 2;23:29. doi: 10.1186/s12937-024-00929-1 (PMC10908051; doi:10.1186/s12937-024-00929-1)
Supplement: Supplementary file 1 — Supplementary Material 1 [file 12937_2024_929_MOESM1_ESM.docx]

Supplemental file 1

**Towards objective measurements of habitual dietary intake patterns: comparing NMR metabolomics and food frequency questionnaire data in a population-based cohort**

Anna Winkvist*^1,2^, Ingegerd Johansson^3^, Lars Ellegård^1,4^, Helen M Lindqvist^1^

**Table S1.** Food group components, scoring algorithm and corresponding FFQ food items included for the indices, scores and clusters used

|  | **HDS** | **rMDS** | **PDI** | **Clusters** |
| --- | --- | --- | --- | --- |
| Food groups included | Favorable foods: fish; fruit (except juice); vegetables (except potatoes); whole grain. Unfavorable foods: red and processed meat; desserts and sweets; sugar-sweetened beverages; fried potatoes | Favorable foods: vegetables excluding potatoes; fruit including nuts and seeds; legumes, fresh and frozen fish excluding fish products and preserved fish, olive oil and cereals. Unfavorable foods: total meat; dairy products; alcohol | Healthful plant foods: whole grains; fruits; vegetables; legumes; vegetable oils; coffee/tea. Unhealthful plant foods: sweetened beverages; refined grains; potato; sweets/desserts. Animal foods: animal fat; dairy; fish/seafood; poultry/red meat; and miscellaneous animal-based foods | Clusters were based on:  high-fat spreads; low-fat spreads; oil; butter in cooking; margarine in cooking; fruit; high-fiber vegetables; milk 0.5%; milk 1.5%; milk 3.0%; cream; high-fat cheese; low-fat cheese; high-fiber cereals; low-fiber cereals; white bread; high-fiber bread; boild potato; fried potato; pasta and rice; fish; red meat; bacon and sausage; chicken; cold cuts; pancake and dumplings; pulses; sweets; sugar and jam; ice cream; cookies; snacks; soda; coffee; tea; beer; wine; spirits; fast food |
| Scoring algorithm | Within each sex, intakes are ranked in ascending quartile ranks for favorable groups and in descending quartile ranks for unfavorable groups. The sum of the quartile ranks yields the score, with a maximum of 24 and higher scores reflecting a healthier diet. | Tertiles of intake, expressed as g*1000/kcal*day, were calculated for favorable foods. For unfavorable foods, similar tertiles were constructed and the scoring was reversed. Alcohol was scored 2 for moderate consumption and 0 for consumption outside of this range. The final score had a maximum of 9, indicating high adherence to a healthy Mediterranean-style diet. | Within each sex, quintiles of frequency of intake/day were constructed. For PDI, participants were assigned 5 points if they were above their fifth quintile of intake of any plant food, 4 points if between the fifth and fourth quintile of intake and so forth. For animal foods the reverse scoring was used. Points for all 15 food groups were summarized to the PDI. | Individuals were predicted to mutually exclusive groups where within-class variance was minimized and between-class variance was maximized. Reported intake per 1,000 kcal was used as input data. |
| Food items in FFQ used | All fruits and berries; root vegetables, lettuce, cabbage, kale; oat-, graham- and rye porridge and fiber-rich muesli; minced meat, stew, steak; bacon and sausage; chicken, hen; hamburgers;  meat, sausage and liverwurst on sandwich;  fried potato and French fries;  high-fat and lean fish, shellfish; candies, chocolate; ; ice cream; sugar, marmalade, jam, honey; cookies, cakes; sodas | Vegetable oils in cooking and as salad dressing; all fruits and berries; root vegetables, lettuce, cabbage, kale; milk, fermented milk 0.5% fat; milk, fermented milk 1.5% fat; milk, fermented milk 3.0% fat; cream, sour cream, crème fraiche; hard cheese 28% fat; hard cheese 17% fat; fiber-rich muesli; corn-flakes etc; minced meat, stew, steak; bacon and sausage; chicken, hen; meat, sausage and liverwurst on sandwich; high-fat and lean fish, shellfish; beans, peas; all types of beer; red and white wine; all types of spirits; | Butter and high-fat margarine; butter used in cooking;vegetable oils in cooking;  all fruits and berries; root vegetables, lettuce, cabbage, kale; milk, fermented milk 0.5% fat; milk, fermented milk 1.5% fat; milk, fermented milk 3.0% fat; cream, sour cream, crème fraiche; hard cheese 28% fat; hard cheese 17%; high-fiber bread, hard and soft; oat-, graham- and rye porridge and fiber-rich muesli; corn-fakes etc; white bread, hard and soft;  minced meat, stew, steak; bacon and sausage; chicken, hen; hamburgers;  meat, sausage and liverwurst on sandwich; pancakes, dumplings;  boiled and mashed potato; fried potato and French fries;  pasta, macaroni and rice; high-fat and lean fish, shellfish; beans, peas; candies, chocolate; ; ice cream; sugar, marmalade, jam, honey; cookies, cakes; sodas;  coffee, boiled and filtered; tea | Butter and high-fat margarine; low-fat margarine; vegetable oils in cooking and as salad dressing; butter used in cooking; margarine used in cooking; all fruits and berries; root vegetables, lettuce, cabbage, kale; milk, fermented milk 0.5% fat; milk, fermented milk 1.5% fat; milk, fermented milk 3.0% fat; cream, sour cream, crème fraiche; hard cheese 28% fat; hard cheese 17% fat; oat-, graham- and rye porridge and fiber-rich muesli; corn-flakes etc; white bread, hard and soft; high-fiber bread, hard and soft; boiled and mashed potato; fried potato and French fries; pasta, macaroni and rice; high-fat and lean fish, shellfish; minced meat, stew, steak; bacon and sausage; chicken, hen; meat, sausage and liverwurst on sandwich; pancakes, dumplings; beans, peas; candies, chocolate; sugar, marmalade, jam, honey; ice cream; cookies, cakes; chips, popcorn, peanuts; sodas; coffee, boiled and filtered; tea; all types of beer; red and white wine; all types of spirits; pizza and hamburger |

HDS, Healthy Diet Score; rMDS, relative Mediterranean Diet Score; PDI, plant based diet index

**Table S2.** Characteristics of food intake in quartiles of indices and scores and in the clusters among participants in the Västerbotten Intervention Programme (n= 1895)

| **Dietary intake pattern** | **Women** | **Men** |
| --- | --- | --- |
| Healthy Diet Score Q1 | High in high-fat spread, margarine, high-fat dairy, white bread, fried potato, red meat, bacon, sausage, fast food, sweets, icecream, sugar, jam, cookies, soda | High in high-fat spread, margarine, white bread, fried potato, bacon, sausage, fast food, sweets, sugar, jam, cookies, snacks, soda, spirits |
| Q4 | High in low-fat spread, oil, butter, fruit, vegetables, lowf-at dairy, cereals, high-fiber bread, boiled potato, fish, pulses, tea, wine | High in low-fat spread, oil, fruit, vegetables, low-fat dairy, high-fiber cereals, high-fiber bread, fish, cold cuts, wine |
| rMDS Q1 | High in high-fat spread, butter, margarine, high-fat dairy, bread, fried potato, red meat, bacon, sausage, cold cuts, fast food, sweets, sugar, jam, cookies, soda | High in high-fat spread, butter, margarine, high-fat dairy, low-fiber cereals, white bread, bacon, sausage, cold cuts, fast food, sweets, sugar, jam, cookies, soda, coffee |
| Q4 | High in low-fat spread, oil, fruit, vegetables, low-fat dairy, high-fiber cereals, fish, chicken, pulses, beer, wine, spirits | High in low-fat spread, oil, fruit, vegetables, low-fat dairy, high-fiber cereals, fish, chicken, pulses, beer, wine, spirits |
| PDI Q1 | High in butter, cream, chicken, cold cuts | High in butter, red meat, bacon, sausage, col dcuts, fish |
| Q4 | High in low-fat spread, oil, fruit, vegetables, low-fat dairy, cereals, high-fiber bread, pulses, sweets, icecream, sugar, jam, cookies, soda, coffee, beer | High in low-fat spread, oil, fruit, vegetables, cereals, high fiber bread, pulses, sweets, sugar, jam, cookies, soda, coffee |
| hPDI Q1 | High in high-fat spread, margarine, butter, high-fat dairy, low-fiber cereals, white bread, fried potato, red meat, bacon, sausage, cold cuts, fast food, sweets, icecream, sugar, jam, cookies, soda | High in high-fat spread, butter, high-fat dairy, white bread, low-fiber cereals, fried potato, red meat, bacon, sausage, fish, cold cuts, fast food, sweets, sugar, jam, cookies, soda, beer, spirits |
| Q4 | High in oil, fruit, vegetables, low-fat dairy, high-fiber cereals, high-fiber bread, pulses, coffee, tea, wine | High in oil, fruit, vegetables, high-fiber cereals, high-fiber bread, coffee, wine |
| uPDI Q1 | High in high-fat spread, butter, margarine, fruit, vegetables, dairy, high-fiber cereals, high-fiber bread, red meat, chicken, cold cuts, fish, pulses, coffee, tea, wine, spirits | High in high-fat spread, butter, margarine, fruit, vegetables, high-fat dairy, high-fiber cereals, high-fiber bread, pasta, rice, red meat, bacon, sausage, cold cuts, chicken, fish, pulses, coffee, tea, beer, wine |
| Q4 | High in low-fat spread, fruit, low-fiber cereals, white bread, sweets, sugar, jam, cookies, soda | High in low-fat spread, margarine, white bread, fast food, sweets, sugar, jam, cookies, soda |
| Cluster 1, Period 1 | High in high-fat spread, high-fat dairy, white bread, sugar, jam, cookies | High in high-fat spread, high-fat dairy, sugar, jam |
| Cluster 2, Period 1 | High in low-fat spread, fruit, low-fat dairy, high-fiber cereals, high-fiber bread, | High in low-fat spread, margarine, low-fat dairy, high-fiber bread, cold cuts |
| Cluster 3, Period 1 | High in cream, red meat, bacon, sausage, fast food, fried potato, pasta, rice, snacks, sweets, soda | High in cream, fried potato, red meat, bacon, sausage, fast food, pasta, rice, soda, coffee, beer |
| Cluster 4, Period 1 | High in oil, pulses, tea | High in oil, low-fat dairy, fruit, vegetables, high-fiber cereals, fish, pulses, tea, wine |

rMDS, relative Mediterranean Diet Score; PDI, plant based diet index; hPDI, healthful plant based diet index; uPDI, unhealthful plant based diet index; Q1, lowest quartile; Q4, highest quartile

**Table S3.** Correlation matrix for indices and scores among women in the Västerbotten Intervention Programme (n=932)

| **Index/score** | **Healthy Diet Score** | **rMED** | **PDI** | **hPDI** | **uPDI** |
| --- | --- | --- | --- | --- | --- |
| Healthy Diet Score | 1 | 0.577^1^  <0.001 | 0.121  <0.001 | 0.615  <0.001 | -0.640  <0.001 |
| rMED |  | 1 | 0.245  <0.001 | 0.596  <0.001 | -0.464  <0.001 |
| PDI |  |  | 1 | 0.320  <0.001 | -0.031  0.031 |
| hPDI |  |  |  | 1 | -0.395  <0.001 |
| uPDI |  |  |  |  | 1 |

1 Spearman correlation coefficient, ρ, and p-value. rMDS, relative Mediterranean Diet Score; PDI, plant based diet index; hPDI, healthful plant based diet index; uPDI, unhealthful plant based diet index

**Table S4.** Correlation matrix for indices and scores among men in the Västerbotten Intervention Programme (n=963)

| **Index/score** | **Healthy Diet Score** | **rMED** | **PDI** | **hPDI** | **uPDI** |
| --- | --- | --- | --- | --- | --- |
| Healthy Diet Score | 1 | 0.522^1^  <0.001 | 0.102  0.001 | 0.584  <0.001 | -0.614  <0.001 |
| rMED |  | 1 | 0.300  <0.001 | 0.453  <0.001 | -0.519  <0.001 |
| PDI |  |  | 1 | 0.301  <0.001 | -0.064  0.048 |
| hPDI |  |  |  | 1 | -0.336  <0.001 |
| uPDI |  |  |  |  | 1 |

1 Spearman correlation coefficient, ρ, and p-value. rMDS, relative Mediterranean Diet Score; PDI, plant based diet index; hPDI, healthful plant based diet index; uPDI, unhealthful plant based diet index
